# Prognostic significance of the pan-immune-inflammation value (PIV) in patients with differentiated thyroid carcinoma

**DOI:** 10.20945/2359-4292-2024-0217

**Published:** 2025-03-24

**Authors:** Yusuf Öztürk, Muhammet Kocabaş, Melia Karaköse, Mustafa Kulaksizoğlu, Feridun Karakurt

**Affiliations:** 1 Department of Endocrinology and Metabolism, Necmettin Erbakan University Faculty of Medicine, Konya, Türkiye

**Keywords:** Pan-immune-inflammation value, differentiated thyroid cancer, distant metastases, high-risk

## Abstract

**Objective:**

The pan-immune-inflammation value (PIV) is a novel inflammatory biomarker for evaluating inflammatory status in patients with cancer. The aim of this study was to evaluate the prognostic value of PIV in patients with differentiated thyroid cancers (DTCs).

**Subjects and methods:**

The study included 376 patients with DTC who underwent curative resection. The PIV was calculated using the formula (neutrophils × monocytes × platelets)/lymphocytes. The TNM stages 3-4 were considered advanced. Patients were also categorized into low-, intermediate-, and high-risk groups according to the AmericanThyroid Association (ATA) risk classification system. Demographic, laboratory, and clinicopathological data were obtained from the patients’ files. The predictive value of PIV on TNM advanced stage, ATA high-risk category, and distant metastases was evaluated using receiver operating characteristic (ROC) curve analysis.

**Results:**

The optimal PIV values for predicting TNM advanced stage, ATA high-risk category, and distant metastases were, respectively, 331.62 (area under the curve [AUC] 0.730, sensitivity 66.7%, specificity 64.8%), 365.52 (AUC 0.822, sensitivity 79.5%, specificity 76.9%), and 357.65 (AUC 0.774, sensitivity 75.2%, specificity 72%). On regression analysis, PIV ≥ 365.52 (odds ratio [OR] 29.150, 95% confidence interval [CI] 8.148-104.290, p < 0.001) and PIV ≥ 357.65 (OR 7.224, 95% CI 1.700-30.693, p = 0.007) emerged as independent risk factors for ATA high-risk category and distant metastases, respectively.

**Conclusion:**

Among patients with DTC, PIV is an independent risk factor for distant metastases and ATA high-risk category. While this finding must be supported by more comprehensive studies, PIV has the potential to be used as a prognostic biomarker in these patients.

## INTRODUCTION

According to current data, thyroid cancer ranks seventh among newly diagnosed cancer cases worldwide (1. Differentiated thyroid cancers (DTCs) account for more than 90% of all thyroid cancers (2. Compared with many other types of cancers, DTC has a favorable prognosis, with a 10-year survival rate above 90% (3. However, approximately one-third of the patients with DTC are at risk of disease recurrence and disease-related death (2.

Two different classification systems are used for the prognostic grading of DTC. The first is the TNM Classification of Malignant Tumors, which is used to estimate the risk of cancer-related death (4. The second is the American Thyroid Association (ATA) risk classification system, which estimates the risk of structural recurrence (2. Both TNM and ATA classifications are based on parameters present at the time of surgery to guide the extent of the initial treatment. The most common sites of distant DTC metastases are the lungs and bones; as in all other cancer types, the development of distant DTC metastases is associated with a poor prognosis (^2,5^), with early identification of metastases being critical for the response to treatment (6.

There is evidence that inflammation can affect proliferation, angiogenesis, and metastasis in cancer tissues through multiple mechanisms and pathways (7. Recent studies and meta-analyses have shown a relationship between cancer prognosis and several inflammatory biomarkers, such as the neutrophil-to- lymphocyte ratio (NLR) (^8^), lymphocyte-to-monocyte ratio (LMR) (^9^), platelet-to-lymphocyte ratio (PLR) (^10^), and systemic immune-inflammation index (SII) (^11^), all of which are obtained from complete blood count (CBC) tests. The pan-immune-inflammation value (PIV) is a novel inflammatory biomarker used to assess inflammation status in cancer patients. It was first reported by Fucà and cols. as a strong predictor of survival outcomes in patients with metastatic colorectal cancer treated with first-line therapy (12. Unlike other inflammatory biomarkers, PIV is calculated from the counts of four peripheral blood cell types. To our knowledge, the prognostic significance of PIV in patients with DTC has not been investigated yet. Based on these considerations, the aim of this study was to investigate the prognostic significance of PIV in patients with DTC.

## SUBJECTS AND METHODS

The study included 376 patients diagnosed with DTC who had undergone curative resection between No- vember 2018 and December 2022. These participants were recruited from the Department of Endocrinol- ogy and Metabolism at Necmettin Erbakan University (NEU) Faculty of Medicine. The Ethics Committee of NEU approved the study protocol (approval ID 2022/4083, approval date December 16, 2022).

The exclusion criteria included patients whose (A) pathology reports from the surgery did not comply with the TNM staging system or (B) data required for the ATA risk classification criteria were missing, in addition to those who had (C) missing parameters in the presurgical CBC test, (D) hematological disease, autoimmune disease (including Hashimoto’s and Graves’ disease), or malignancy other than thyroid cancer, (E) prior surgery for thyroid cancer, or (F) used medications related to inflammation (*e.g.*, glucocorticoids).

Demographic data and clinicopathological charac- teristics were obtained from the patients’ medical re- cords and included age, sex, histopathological subtype, tumor diameter, number of tumor foci, presence of vascular, extrathyroidal, capsular, lymphatic and peri- neural invasion, presence of surgical margin involve- ment and lymph node metastasis status. The absolute neutrophil count (ANC), absolute lymphocyte count (ALC), absolute monocyte count (AMC), and abso- lute platelet count (APC) were estimated from CBC tests obtained up to 1 month before surgery. The PIV value was calculated using the formula (neutrophils × monocytes × platelets)/lymphocytes. Additionally, the monocyte-to-lymphocyte ratio (MLR), NLR, and PLR values were calculated using the formulas monocytes/ lymphocytes, neutrophils/lymphocytes, and plate- lets/lymphocytes, respectively. Histopathological data were obtained from institutional routine pathology reports. The tumors’ TNM stage was determined us- ing the TNM staging system, 8th edition (13. The TNM stages 1 and 2 were classified as early stages, and stages 3 and 4 were classified as advanced stages. Pa- tients were categorized as having low, intermediate, or high risk for recurrence or persistent disease accord- ing to the ATA risk classification system using clini- copathological features. The distant metastasis status of the patients was recorded. Distant metastases were detected by imaging methods (computed tomogra- phy, magnetic resonance imaging, bone scintigraphy, or 18F-fluorodeoxyglucose positron emission tomog- raphy-computed tomography [18F-FDG PET/CT]). Patients with TNM advanced-stage disease, ATA high- risk status, and distant metastases were considered to have a poor prognosis.

### Statistical analysis

The statistical analysis was performed using the software SPSS 22.0 (Statistical Package for Social Sciences; IBM Corp., Armonk, NY, USA). The Kolmogorov-Smirnov test was used to determine the normality of the variables’ distribution. Continuous variables with normal distribution were presented as mean ± standard deviation, while those without normal distribution were presented as median (minimum–maximum) values. The cutoff values for PIV, ALC, ANC, AMC, APC, MLR, NLR, and PLR for predicting poor prognosis were determined by receiver operating characteristic (ROC) curve analysis. Univariate and stepwise multivariate analyses were performed using a logistic regression model to analyze prognostic factors. For differences, p values < 0.05 were considered statistically significant.

## RESULTS

Of the 376 patients included in this study, 288 (76.6%) were female and 88 (23.6%) were male. Their mean age at diagnosis was 45.1 ± 13.90 years. The most common histopathological type was papillary carcinoma, which was diagnosed in 342 (^91^)%) of the patients. Overall, 15 (^4^)%) patients had advanced-stage tumors according to the TNM classification, 39 (10.4%) had high-risk disease according to the ATA risk classification, and 20 (5.3%) had distant metastases (Table 1).

**Table 1:** Clinical and histopathological characteristics of patients with differentiated thyroid cancer (DTC)

Variables	n = 376
Sex	
Female, n (%)	288 (76.6)
Male, n (%)	88 (23.6)
Age, years	45.10 ± 13.90
Histopathological type	
Papillary microcarcinoma, n (%)	107 (28.5)
Papillary carcinoma, n (%)	235 (62.5)
Follicular carcinoma, n (%)	21 (5.6)
Oncocytic carcinoma, n (%)	13 (3.5)
Tumor size, mm	19.06 ± 15.10
Number of tumor foci	
Unifocal, n (%)	234 (62.8)
Multifocal, n (%)	142 (37.8)
Number of patients with positive histopathological prognostic factors	
Positive surgical margins, n (%)	42 (11.2)
Angioinvasion, n (%)	43 (11.4)
Extrathyroidal extension, n (%)	76 (20.2)
Capsular invasion, n (%)	63 (16.8)
Lymphatic invasion, n (%)	93 (24.7)
Perineural invasion, n (%)	18 (4.8)
Lymph node involvement, n (%)	121 (32.2)
ANC (1,000 cells/mm^3^)	4.646 ± 1.582
ALC (1,000 cells/mm^3^)	2.336 ± 0.777
AMC (1,000 cells/mm^3^)	0.517 ± 0.194
APC (1,000 cells/mm^3^)	281.752 ± 62.630
PIV	318.080 ± 223.097
MLR	0.235 ± 0.102
NLR	2.160 ± 1.085
PLR	131.621 ± 48.481
TNM classification 8th edition, n (%)	
I	324 (86.2)
II	37 (9.8)
III	2 (0.5)
IV	13 (3.5)
ATA classification category	
Low, n (%)	192 (51.1)
Intermediate, n (%)	145 (38.6)
High, n (%)	39 (10.4)
Distant metastases, n (%)	20 (5.3)
Lung, n (%)	17 (4.5)
Bone, n (%)	9 (2.4)

All values are presented as mean ± standard deviation or n (%). Abbreviations: ALC, absolute lymphocyte count; AMC, absolute monocyte count; ANC, absolute neutrophil count; APC, absolute platelet count; ATA, American Thyroid Association; MLR, monocyte-to-lymphocyte ratio; NLR, neutrophil-to-lymphocyte ratio; PIV, pan-immune-inflammation value; PLR, platelet-to-lymphocyte ratio; TNM, TNM Classification of Malignant Tumors.

**Figure 1:**
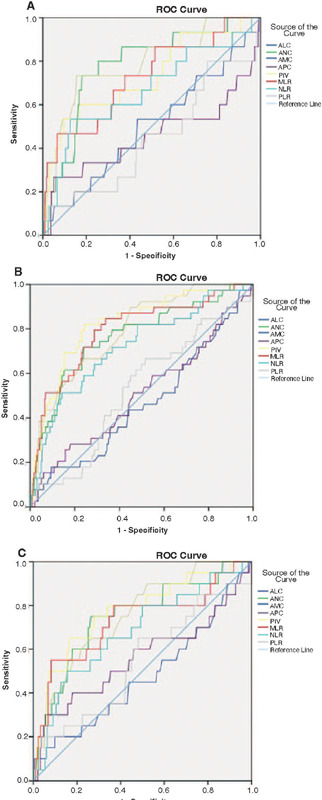
Receiver operating characteristic (ROC) curve analysis of the predictive values of absolute lymphocyte count (ALC), absolute neutrophil count (ANC), absolute monocyte count (AMC), absolute platelet count (APC), pan-immune-inflammation value (PIV), monocyte-to-lymphocyte ratio (MLR), neutrophil-to-lymphocyte ratio (NLR), and platelet-to- lymphocyte ratio (PLR) in patients with differentiated thyroid cancer (DTC) with (A) TNM advanced stage, (B) ATA high-risk category, (C) or/and distant metastases.

We assessed the predictive value of PIV for identifying TNM advanced stages, ATA high-risk category, and distant metastases. According to the ROC curve analysis, the optimal PIV cutoff values for TNM advanced stages, ATA high-risk category, and distant metastases were, respectively, 331.62 (area under the curve [AUC] 0.730, sensitivity 66.7%, specificity 64.8%), 365.52 (AUC 0.822, sensitivity 79.5%, specificity 76.9%), and 357.65 (AUC 0.774, sensitivity 75.0%, specificity 72.2%) (Table 2 and Figure 1).

**Table 2:** Receiver operating characteristic (ROC) curve analysis of the predictive value of absolute lymphocyte (ALC), neutrophil (ANC), monocyte (AMC), and platelet (APC) counts, pan-immune-inflammation value (PIV), and monocyte-to-lymphocyte (MLR), neutrophil-to-lymphocyte ratio (NLR), and platelet-to-lymphocyte (PLR) ratios

	AUC	95% CI	Cutoff value	Sensitivity	Specificity	P value
TNM advanced stage						
ALC	0.502	0.341-0.664	2.28	53.3%	56.5%	0.975
ANC	0.783	0.668-0.898	5.36	80.0%	74.5%	0.000*
AMC	0.787	0.660-0.913	0.665	73.3%	84.5%	0.000*
APC	0.455	0.259-0.651	279.5	46.7%	53.2%	0.557
PIV	0.730	0.584-0.877	331.62	66.7%	64.8%	0.002*
MLR	0.727	0.586-0.868	0.246	66.7%	67.6%	0.003*
NLR	0.669	0.510-0.828	2.29	60.0%	68.7%	0.027*
PLR	0.446	0.282-0.610	129.61	46.7%	54.6%	0.479
ATA high-risk category						
ALC	0.464	0.364-0.564	2.185	46.2%	47.8%	0.461
ANC	0.765	0.680-0.849	5.335	71.8%	76.0%	0.000
AMC	0.791	0.720-0.861	0.549	69.2%	66.5%	0.000
APC	0.505	0.402-0.609	279.5	51.3.2%	53.7%	0.915
PIV	0.822	0.753-0.892	365.52	79.5%	76.9%	0.000*
MLR	0.793	0.712-0.873	0.249	74.4%	72.1%	0.000
NLR	0.719	0.631-0.808	2.23	66.7%	68.0%	0.000
PLR	0.525	0.431-0.619	129.61	56.4%	55.8%	0.610
Distant metastases						
ALC	0.476	0.333-0.619	2.115	50.0%	43.3%	0.716
ANC	0.748	0.631-0.864	5.360	70.0%	74.7%	0.000
AMC	0.754	0.652-0.857	0.595	65.0%	73.3%	0.000
APC	0.560	0.402-0.717	283.5	55.0%	56.2.%	0.367
PIV	0.774	0.659-0.889	357.65	75.0%	72.2%	0.000*
MLR	0.727	0.591-0.862	0.246	70.0%	68.3%	0.001
NLR	0.682	0.554-0.809	2.237	65.0%	66.0%	0.006
PLR	0.543	0.403-0.682	129.61	55.0%	55.1%	0.521

Abbreviations: ALC, absolute lymphocyte count; AMC, absolute monocyte count; ANC, absolute neutrophil count; APC, absolute platelet count; AUC, area under the curve; CI, confidence interval; MLR, monocyte-to-lymphocyte ratio; NLR, neutrophil-to-lymphocyte ratio; PIV, pan-immune-inflammation value; PLR, platelet-to-lymphocyte ratio; ROC, receiver operating characteristic.

* *P* values less than 0.05 were considered statistically significant.

On regression analysis, age (OR 1.120, 95% confidence interval [CI] 1.052-1.192, p = 0.000), tumor size (OR 1.027, 95% CI 1.001-1.054, p = 0.045), and AMC (OR 13.220, 95% CI 3.389-51.563, p = 0.000) emerged as independent risk factors for TNM advanced stage (Table 3). Additionally, age (OR 1.052, 95% CI 1.015-1.091, p = 0.005), tumor size (OR 1.093, 95% CI 1.057-1.131, p < 0.001), lymph node involvement (OR 5.793, 95% CI 1.976-16.985, p = 0.001), capsular invasion (OR 3.714, 95% CI 1.197-11.518, p = 0.023), and PIV ≥ 365.52 (OR 29.150, 95% CI 8.148-104.290, p < 0.001) emerged as independent risk factors for ATA high-risk category (Table 4), while age (OR 1.124, 95% CI 1.054-1.199, p = 0.000), tumor size (OR 1.078, 95% CI 1.040=1.118, p < 0.001), and PIV ≥ 357.65 (OR 7.224, 95% CI 1.700-30.693, p = 0.007) emerged as independent risk factors for distant metastases (Table 5).

**Table 3:** Evaluation of prognostic factors for TNM advanced stage

	Univariate analysis		Multivariate analysis	
	OR (^95^)% CI)	P	OR (^95^)% CI)	P
Sex	3.025 (1.065-8.593)	0.038		
Age	1.148 (1.085-1.215)	<0.001*	1.120 (1.052-1.192)	0.000*
Tumor size	1.054 (1.031-1.078)	<0.001*	1.027 (1.001-1.054)	0.045*
Number of tumor foci	0.399 (0.111-1.440)	0.161		
Positive surgical margins	4.378 (1.420-13.501)	0.010		
Angioinvasion	0.071 (0.912-9.885)	0.071		
Extrathyroidal extension	4.924 (1.726-14.046)	0.003		
Capsular invasion	11.623 (3.821-35.351)	<0.001*		
Lymphatic invasion	2.798 (0.986-7.939)	0.053		
Perineural invasion		0.998		
Lymph node involvement	0.768 (0.239-2.463)	0.657		
ANC ≥ 5.360	11.696 (3.229-42.365)	0.000*		
AMC ≥ 0.665	14.978 (4.605-48.710)	0.000*	13.220 (3.389-51.563)	0.000*
PIV ≥ 331.62	3.685 (1.233-11.016)	0.020	2.876 (0.752-10.995)	0.123
MLR ≥ 0.246	3.950 (1.321-11.815)	0.014		
NLR ≥ 2.29	3.224 (1.121-9.273)	0.030		

Abbreviations: AMC, absolute monocyte count; ANC, absolute neutrophil count; CI, confidence interval; MLR, monocyte-to-lymphocyte ratio; NLR, neutrophil-to-lymphocyte ratio; OR, odds ratio; PIV, pan-immune-inflammation value.

* *P* values less than 0.05 were considered statistically significant.

**Table 4:** Evaluation of prognostic factors for ATA high-risk category

	Univariate analysis		Multivariate analysis	
	OR (^95^)% CI)	P	OR (^95^)% CI)	P value
Sex	4.701 (2.372-9.318)	<0.001*		
Age	1.080 (1.049-1.112)	<0.001*	1.052 (1.015-1.091)	0.005*
Tumor size	1.085 (1.059-1.110)	<0.001*	1.093 (1.057-1.131)	0.000*
Number of tumor foci	0.708 (0.346-1.446)	0.343		
Positive surgical margins	2.300 (0.979-5.404)	0.056		
Angioinvasion	5.117 (2.383-10.985)	<0.001*		
Extrathyroidal extension	5.982 (2.992-11.958)	<0.001*		
Capsular invasion	7.197 (3.557-14.562)	<0.001*	3.714 (1.197-11.518)	0.023*
Lymphatic invasion	2.994 (1.517-5.910)	0.002*		
Perineural invasion	1.084 (0.240-4.903)	0.916		
Lymph node involvement	2.220 (1.136-4.337)	0.020	5.793 (1.976-16.985)	0.001*
ANC ≥ 5.335	8.045 (3.835-16.876)	0.000*		
AMC ≥ 0.549	4.401 (2.150-9.010)	0.000*		
PIV ≥ 365.52	12.867 (5.682-29.139)	<0.001*	29.150 (8.148-104.290)	0.000*
MLR ≥ 0.249	7.280 (3.416-15.516)	0.000*		
NLR ≥ 2.237	4.241 (2.098-8.574)	0.000*		

Abbreviations: AMC, absolute monocyte count; ANC, absolute neutrophil count; CI, confidence interval; MLR, monocyte-to-lymphocyte ratio; NLR, neutrophil-to-lymphocyte ratio; OR, odds ratio; PIV, pan-immune-inflammation value.

* *P* values less than 0.05 were considered statistically significant.

**Table 5:** Evaluation of prognostic factors for distant metastases

	Univariate analysis		Multivariate analysis	
	OR (^95^)% CI)	P	OR (^95^)% CI)	P
Sex	4.429 (1.771-11.072)	0.001*		
Age	1.172 (1.106-1.241)	0.000*	1.124 (1.054-1.199)	0.000*
Tumor size	1.099 (1.065-1.134)	0.000*	1.078 (1.040-1.118)	0.000*
Number of tumor foci	0.171 (0.039-0.750)	0.019		
Positive surgical margins	2.874 (0.988-8.360)	0.053		
Angioinvasion	4.786 (1.794-12.770)	0.002*		
Extrathyroidal extension	4.394 (1.757-10.986)	0.002*		
Capsular invasion	8.971 (3.496-23.021)	0.000*		
Lymphatic invasion	3.289 (1.324-8.174)	0.010*		
Perineural invasion		0.988		
Lymph node involvement	0.910 (0.341-2.429)	0.850		
ANC ≥ 5.360	6.896 (2.573-18.481)	0.000*		
AMC ≥ 0.595	5.102 (1.976-13.172)	0.001*		
PIV ≥ 357.65	7.788 (2.757-21.997)	0.000*	7.224 (1.700-30.693)	0.007*
MLR ≥ 0.246	4.706 (1.764-12.558)	0.002*		
NLR ≥ 2.237	3.607 (1.402-9.277)	0.008*		

Abbreviations: AMC, absolute monocyte count; ANC, absolute neutrophil count; CI, confidence interval; MLR, monocyte-to-lymphocyte ratio; NLR, neutrophil-to-lymphocyte ratio; OR, odds ratio; PIV, pan-immune-inflammation value.

* *P* values less than 0.05 were considered statistically significant.

## DISCUSSION

The present study evaluated the prognostic value of PIV – a new biomarker derived from CBC parameters – in patients with DTC. The results of the study revealed that PIV is an independent risk factor for distant metastases and ATA high-risk category in these patients. Additionally, age and tumor size emerged as independent risk factors for TNM advanced stage, ATA high-risk category, and distant metastases.

Previous studies have suggested that circulating immune cells play a significant role in the long-term prognosis of some types of cancer (^14,15,16,17^). Studies have also shown that the body’s overall immune sys- tem significantly influences cancer development and control by influencing the tumor microenvironment (18. Different types of immune cells exist in the tu- mor microenvironment and have significant effects on tumor progression (19. There is ample evidence im- plicating an antitumor immune response mediated by cytotoxic T lymphocytes and NK cells (18. In contrast, tumor-associated neutrophils, platelets, monocyte- derived tumor-associated macrophages, and myeloid- derived suppressor cells play important roles in promoting tumor progression and metastasis (^18,20,21,22,23^). A meta-analysis focused on examining the potential of inflammatory markers such as NLR, LMR, and PLR as predictors of thyroid malignancies, with emphasis on their systemic effects (24. In this meta-analysis, which included 7,599 patients with DTC, no relationship was shown between inflammatory biomarkers – NLR, PLR, and LMR – and disease-free survival (24. A study in- vestigating the potential diagnostic and prognostic value of systemic inflammatory markers in anaplastic thyroid cancer (ATC) and DTC found that the neutro- phil-monocyte-platelet-to-lymphocyte ratio (NMPLR) has an excellent potential in the differential diagnosis of ATC from advanced DTC and survival prediction in refractory thyroid cancer (25. In the present study, we found that PIV may be a valuable biomarker in pre- dicting the risk of recurrence and distant metastases in patients with DTC. Our study also revealed that PIV is more valuable than MLR, NLR, and PLR in predicting the risk of recurrence and distant metastases.

The majority of patients with DTC have a favorable prognosis. However, considering that 2% of patients have distant metastases at the time of diagnosis, DTC may occasionally show aggressive features (26. Although distant metastases are not a common problem in DTC, and the prognosis of patients with this condition is more favorable than those with other cancers (^27^), they can negatively affect survival (^28,29,30^). Therefore, it is essential to identify this patient group for effective prognostic evaluation and therapeutic decisions. A meta-analysis evaluating the clinicopathological risk factors associated with distant metastases in patients with DTC found that various clinicopathological parameters – such as male sex, age ≥ 45 years, tumor size ≥ 4 cm, multifocality, vascular invasion, extrathyroidal extension, and lymph node involvement – were associated with a higher frequency of distant metastases (31. In our study, we found that the frequency of distant metastases was around 5%. In the univariate regression analysis, sex, age, tumor size, angioinvasion, extrathyroidal extension, capsular invasion, lymphatic invasion, and PIV emerged as risk factors for distant metastases, while in the multivariate regression analysis, age, tumor size, and PIV emerged as independent risk factors. The results of our study indicate that PIV has the potential to predict distant metastases in patients with DTC.

Scoring systems proposed to classify thyroid cancers according to recurrence risk are mainly based on clinicopathological features. Patients with DTC are classified as having low (<5%), intermediate (^5^)%-20%), or high (>20%) risk of recurrence according to the ATA risk classification (^2,32^). In the intermediate-risk group, the risk of recurrence varies greatly between patients. Notably, PIV – which was found to be an independent predictor of high risk in our study – may also guide us in predicting recurrence in the intermediate-risk group. As an inflammatory marker, PIV may help us identify high-risk patients at the time of diagnosis and more accurately guide preoperative therapeutic decisions.

Of note, PIV can be easily calculated using CBC parameters and is very cost-effective and simple to obtain at any center. Therefore, PIV has the potential to be used for prognosis assessment in patients with DTC. Despite these findings, our results should be interpreted with caution due to some limitations of our study. First, as a retrospective study conducted in a single institution, selection bias is inevitable. Second, although we excluded any patient with a history of diseases that may affect leukocytes or using medications such as glucocorticoids, the results of circulating cell counts may be affected by other unknown or undetectable factors, such as different medications. Finally, the relatively small sample size in our study weakens the power of the statistical analysis.

In conclusion, we found that PIV calculated from preoperative CBC parameters is an independent risk factor for distant metastases and ATA high-risk category in patients with DTC. Based on the results of our study, we believe that PIV has the potential to be used to predict distant metastases and ATA high-risk category among patients with DTC. However, more comprehensive multicenter studies with a prospective design are needed to confirm these results.
